# Low-Temperature Soft-Cover-Assisted Hydrolysis Deposition of Large-Scale TiO_2_ Layer for Efficient Perovskite Solar Modules

**DOI:** 10.1007/s40820-018-0203-7

**Published:** 2018-04-30

**Authors:** Jinjin He, Enbing Bi, Wentao Tang, Yanbo Wang, Xudong Yang, Han Chen, Liyuan Han

**Affiliations:** 1 State Key Laboratory of Metal Matrix Composites, School of Material Science and Engineering, Shanghai Jiao Tong University, 800 Dong Chuan Road, Shanghai, 200240 People’s Republic of China; 2 Materials Genome Initiative Center, Shanghai Jiao Tong University, 800 Dong Chuan Road, Shanghai, 200240 People’s Republic of China

**Keywords:** Low-temperature, TiO_2_, Large-scale, Soft-cover-assisted hydrolysis deposition, Perovskite solar cell

## Abstract

Perovskite solar cells with TiO_2_ electron transport layers exhibit power conversion efficiency (PCE) as high as 22.7% in single cells. However, the preparation process of the TiO_2_ layer is adopted by an unscalable method or requires high-temperature sintering, which precludes its potential use for mass production of flexible devices. In this study, a scalable low-temperature soft-cover-assisted hydrolysis (SAH) method is presented, where the precursor solution is sandwiched between a soft cover and preheated substrate to form a closed hydrolysis environment. Compact homogeneous TiO_2_ films with a needle-like structure were obtained after the hydrolysis of a TiCl_4_ aqueous solution. Moreover, by careful optimization of the TiO_2_ fabrication conditions, a high PCE of 14.01% could be achieved for a solar module (4 × 4 cm^2^) prepared using the SAH method. This method provides a novel approach for the efficient scale-up of the low-temperature TiO_2_ film growth for industrial applications.

**Electronic supplementary material:**

The online version of this article (10.1007/s40820-018-0203-7) contains supplementary material, which is available to authorized users.

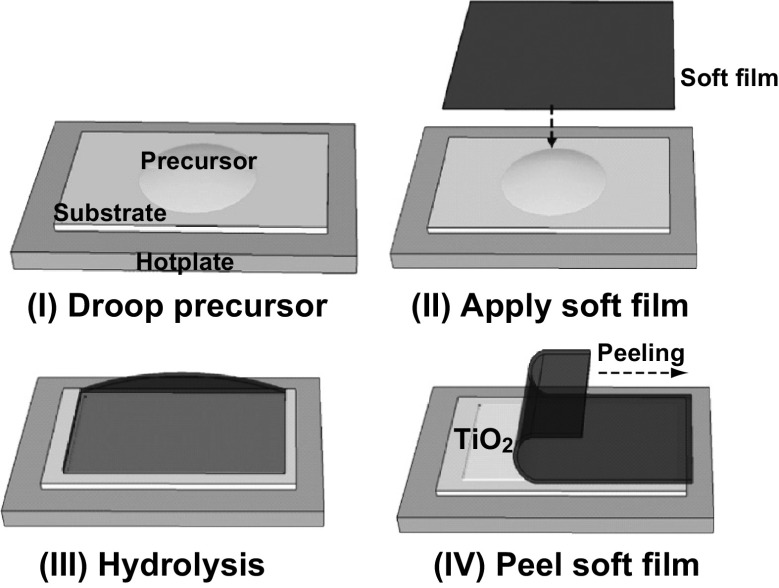

## Highlights

- A simple soft-cover-assisted hydrolysis method to prepare TiO_2_ films at a low temperature is proposed.
- Compact homogeneous large-area TiO_2_ films with a needle-like morphology were obtained.
- A solar module fabricated with as-prepared TiO_2_ films as electron transfer layers exhibited a power conversion efficiency of 14.01%.

## Introduction

Perovskite solar cells (PSCs) have been demonstrated to be next-generation photovoltaic devices that meet future energy-generation demands owing to their high power conversion efficiency (PCE), low cost, simple solution-based preparation, lightweight, and flexibility [1–6]. Conventional PSCs using TiO_2_ as the electron transport material have exhibited high PCEs [7–10]. However, the high-temperature processing of the TiO_2_ layer makes their manufacture more complex and hampers the development of lightweight and flexible substrates. To overcome this limitation, several deposition techniques to fabricate TiO_2_ films at low temperatures have been successfully implemented, such as spin coating (SC) [11–13], atomic layer deposition (ALD) [14, 15], sputtering [16–18], chemical bath deposition [19, 20], and electron-beam evaporation [21].

Among these techniques, SC is the principal method for low-temperature TiO_2_ film preparation. A PSC based on a TiO_2_ layer prepared by SC chlorine-capped TiO_2_ colloidal nanocrystal solutions has achieved a PCE of 19.5%, with an active area of 1.1 cm^2^, which is the highest PCE reported for PSCs prepared at low temperatures [22]. Non-SC methods, including ALD, sputtering, chemical bath deposition, and electron-beam evaporation, are also applicable for controllable TiO_2_ film deposition and have achieved PCEs up to 19% for active areas smaller than 1 cm^2^ [7]. However, it is obvious that neither SC nor above non-SC methods are suitable for a large-scale PSC manufacture owing to their inherent limitations [23, 24]. Therefore, the investigation of simple TiO_2_-layer preparation methods involving a large area and low temperatures is necessary for industrial applications.

In this study, we report a simple low-temperature soft-cover-assisted hydrolysis (SAH) method, where a soft polyimide (PI) film is used to cover a TiCl_4_ aqueous solution spread on a preheated substrate. Compact and homogeneous large-area TiO_2_ films with a needle-like morphology (SAH–TiO_2_) were obtained after the hydrolysis. Moreover, a solar module (4 × 4 cm^2^) with an SAH–TiO_2_ layer as an electron transport layer using the SAH method exhibited a PCE of 14.01% in a conventional device configuration at low temperatures. Therefore, the proposed SAH technology provides a novel non-SC route to the deposition of large-area TiO_2_ films for industrial applications.

## Experimental Methods

### Materials and Reagents

All chemicals were used as received. PbI_2_ (99%), *N*,*N*-dimethylformamide (anhydrous), dimethyl sulfoxide, and chlorobenzene were purchased from Sigma Aldrich. Methylammonium iodide (98%) was obtained from Tokyo Chemical Industry Co. Ltd. Titanium tetrachloride (TiCl_4_) was purchased from Alfa Aesar. A low-temperature TiO_*x*_ SC solution was purchased from Shanghai MaterWin New Materials Co., Ltd.

### Preparation of an SAH–TiO_2_ Layer

The substrates were cleaned by a detergent followed by sequential ultrasonic washing in deionized water, ethanol, and acetone (for 30 min in each of them); they were then dried under nitrogen gas. After 15 min of oxygen plasma treatment, the substrates were preheated at 75 °C for 10 min on a heating plate. Different concentrations (0.1–0.6 M) of 25 μL cm^−2^ TiCl_4_ aqueous solution were added at the centers of the substrates, and a piece of a soft film, used as the soft cover, was immediately placed on the precursor. The covered film was peeled off after the hydrolysis for 20 min, followed by washing with deionized water and ethanol; the obtained film was then dried in air.

### Fabrication of PSCs and Modules

A perovskite precursor solution (90 μL) comprising 1.3 M PbI_2_ and CH_3_NH_3_I (1:1/n:n) in *N*,*N*-dimethylformamide and dimethyl sulfoxide (4:1/v:v) was spread on the SAH–TiO_2_ film using a consecutive two-step SC process at 1000 and 5000 rpm for 12 and 30 s, respectively. Chlorobenzene (500 μL) was dropped on top of the substrates during the second SC step, 20 s before the end of the procedure, followed by annealing at 100 °C for 10 min. A precursor solution of the hole transport layer (HTL) was prepared by dissolving 72.3 mg spiro-OMeTAD, 28.8 μL 4-tert-butylpyridine, and 17.5 μL lithium bis(trifluoromethylsulphonyl)imide acetonitrile solution (520 mg mL^−1^) into 1 mL chlorobenzene. The HTL was then deposited on perovskite by SC at 3000 rpm for 30 s. An 80-nm-thick Au electrode was then thermally evaporated on top of the device to form the back contact. The active area of the device was fixed to 1.02 cm^2^.

For the solar module, the fabricating process was similar to that of small solar cells; the laser scribing patterning process was consistent with that reported previously [25]. In brief, 400 μL TiCl_4_ precursor solution was used for a 6 × 6 cm^2^ substrate. For standard control samples, 1.5 mL TiO_*x*_ solution was spin-coated onto a cleaned fluorine-doped tin oxide (FTO) substrate with the same area at 3000 rpm for 30 s. The laser scribing process included scribing on a 4 × 4 cm^2^ FTO/SAH–TiO_2_ layer using a 1064-nm laser. A pulse laser with energy of 30 μJ, spot size of 25 μm, pulse frequency of 30 kHz, and low scribing speed of 50 mm s^−1^ was used to scribe FTO/SAH–TiO_2_ substrates with a line width of 100 μm. We then used pulse energy of 10 μJ, spot size of 25 μm, pulse frequency of 20 kHz, and scribing speed of 20 mm s^−1^ to scribe perovskite/TiO_2_ and HTL with a line width of 300 μm. Finally, pulse energy of 10 μJ, spot size of 25 μm, pulse frequency of 35 kHz, and scribing speed of 80 mm s^−1^ were applied to scribe the Au electrode with a line width of 100 μm.

### Measurement and Characterization

Current–voltage characteristics were measured using a solar simulator (Oriel Class A, 91195A; Newport) and source meter (2400 series; Keithley) at 100 mW cm^2^ and AM 1.5 G illumination. The simulated light intensity was calibrated with a silicon photodiode. The *J*–*V* curves were measured in the reverse (from 1.2 to − 0.2 V) or forward (from − 0.2 to 1.2 V) scanning modes [26]. The voltage step was fixed at 10 mV, and the delay time (delay at each voltage step before the measurement of the current) was fixed at 50 ms. Monochromatic incident photon-to-current conversion efficiency spectra were measured using a monochromatic incident light (1 × 10^16^ photons cm^−2^) in the direct-current mode (CEP-2000BX; Bunko-Keiki). The morphologies and thicknesses of the films were investigated using a field-emission scanning electron microscope (SEM) (JSM-7800F; JEOL). The microstructure of the product was investigated by a field-emission transmission electron microscope (TEM) (JEM-2100F, JEOL). X-ray diffraction (XRD) patterns of the samples were recorded using an X-ray diffractometer (Ultima IV; Rigaku) with Cu K radiation of 1.54 Å and speed of 21° min^−1^. Ultraviolet–visible (UV–Vis) absorption spectra were recorded using a spectrophotometer (UV-2450; Shimadzu) in a wavelength range of 200–800 nm at room temperature. X-ray spectroscopy measurements were performed using an ESCALAB 250Xi spectrometer (Thermo Scientific).

## Results and Discussion

**Fig. 1:**
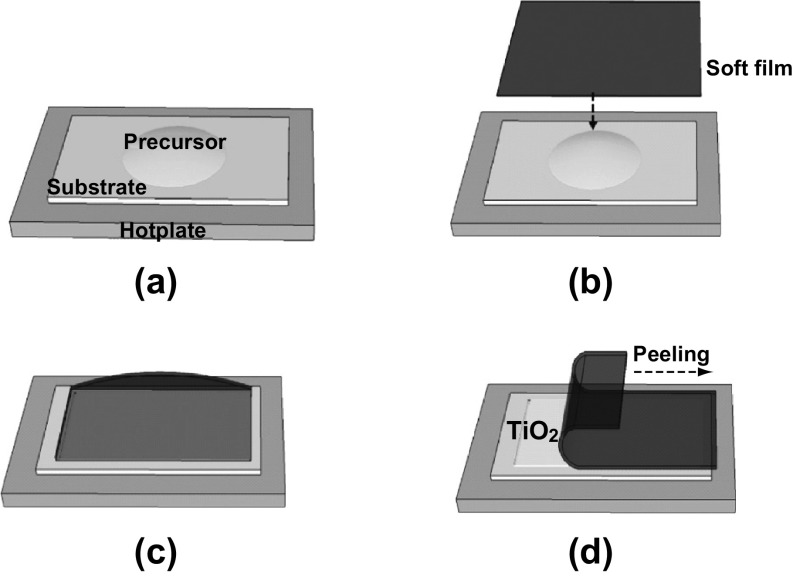
Schematics of the steps in the SAH

Schematics of the steps involved in the SAH are shown in Fig. 1. A certain amount of the TiCl_4_ precursor solution was dropped onto the preheated substrate at 75 °C (Fig. 1a), and a piece of the soft film was used to cover the liquid precursor (Fig. 1b). The precursor solution spreads out into a liquid film through the capillary attraction between the precursor solution and soft film (Fig. 1c). A relatively closed environment was formed to prevent rapid evaporation of the solvent into the air. The soft film was then peeled off by a programmed mechanical hand after 20 min of hydrolysis; consequently, a raw TiO_2_ film was obtained (Fig. 1d). The deposited substrate was rinsed with water and ethanol successively to remove any loosely bound materials or unreacted precursor solution. According to the features of solution-based underlayer preparation techniques, the flatness of the TiO_2_ film in the SAH significantly depended on the uniform liquid film formed during the precursor spreading. This would be related to the wettability of the capped soft film to the precursor solution. A larger wettability implies a larger capillarity between the soft film and solution, which helped form a thin uniform liquid film [27]. The aqueous solution was considered to be the main component used for the hydrolysis; therefore, the candidate soft film should have good wettability with the aqueous solution. Some of the available soft films, such as polyethylene (PE), polyethylene terephthalate (PET), polyvinyl chloride (PVC), and PI films were selected and their contact angles were measured by dropping the aqueous solution onto their surfaces at room temperature in ambient air. As shown in Fig. S1, the average contact angles of the PE, PET, PVC, and PI films were 86°, 81°, 78°, and 52°, respectively. The contact angle of the PI film was significantly smaller than those of the other films; therefore, PI exhibited an excellent wettability with the aqueous solution and was selected as the soft cover for the SAH.

**Fig. 2:**
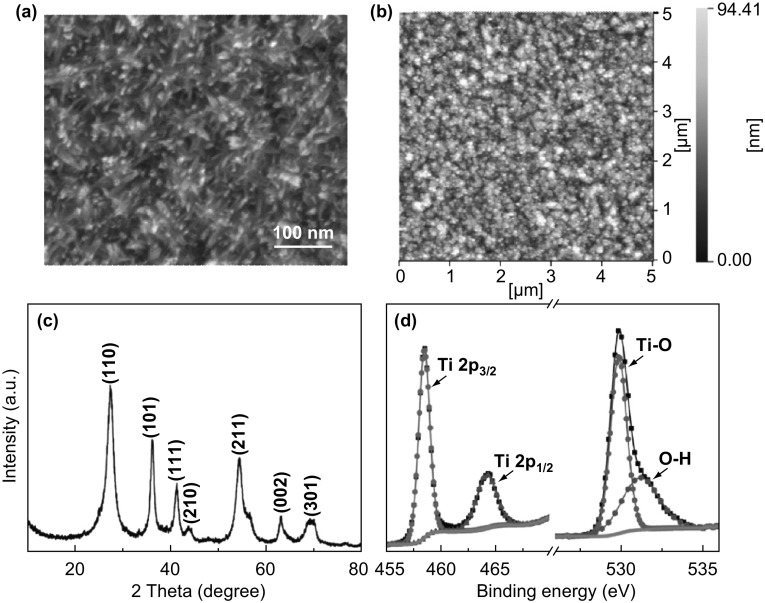
**a** SEM, **b** AFM images of the TiO_2_ films obtained by SAH with a 0.4-M TiCl_4_ precursor solution. **c** XRD patterns, **d** XPS results of the TiO_2_ material scratched from the TiO_2_ film

SEM images of the TiO_2_ layers on the FTO substrates formed by the SAH using a 0.4-M TiCl_4_ aqueous solution are shown in Fig. 2a. The needle-like TiO_2_ completely covered the surface of the FTO substrate to form a continuous TiO_2_ layer; the resulting SAH–TiO_2_ film showed good optical transparency with transmittance larger than 70% in the visible region (Fig. S2) and uniform current distributions (Fig. S3). Moreover, the size and morphology of the as-prepared TiO_2_ product scratched from the TiO_2_ film were analyzed by TEM, as shown in Fig. S3. The TEM images revealed that several nanoneedles were stacked together to form a flower-like structure. The TiO_2_ film was also analyzed by atomic force microscopy (AFM) to provide further insights and obtain quantitative information about the film roughness (Fig. 2b). The average root-mean-square roughness for an area of 5 × 5 μm^2^ was 14.7 nm, which was propitious for the deposition of perovskite [28]. The morphology of the perovskite films based on the SAH–TiO_2_ film is presented in Fig. S4. The surface exhibited a uniform morphology with dense grains. The entire film was composed of a homogeneous well-crystallized perovskite layer, with crystalline grain lengths on the order of hundreds of nanometers. This may have been induced by the smooth morphology of the SAH–TiO_2_ film, which was beneficial for the growth of perovskite layers. More details about the crystal structure of the product are shown in Fig. 2c. The main diffraction peaks were observed at 27.45°, 36.08°, 41.33°, 43.82°, 54.35°, 62.74°, and 69.01°, corresponding to the (110), (101), (111), (210), (211), (002), and (301) planes, respectively, agreeing well with the rutile phase (JCPDS card No. 21-1276) [29]. X-ray photoelectron spectroscopy (XPS) was used to investigate the chemical composition of the TiO_2_ film. The Ti 2*p*_3/2_, Ti 2*p*_1/2_, and O 1*s* characteristic peaks of the TiO_2_ film are shown in Fig. 2d. The Ti 2*p* spectra were identical to the Ti 2*p*_3/2_ and 2*p*_1/2_ spectra, with peaks centered at binding energies of 458.9 and 464.7 eV, respectively, typical for Ti^4+^ states. The corresponding O 1*s* spectra showed two major peaks at 529.9 and 532.6 eV, assigned to the lattice oxygen and surface bridging oxygen, respectively [30].

**Fig. 3:**
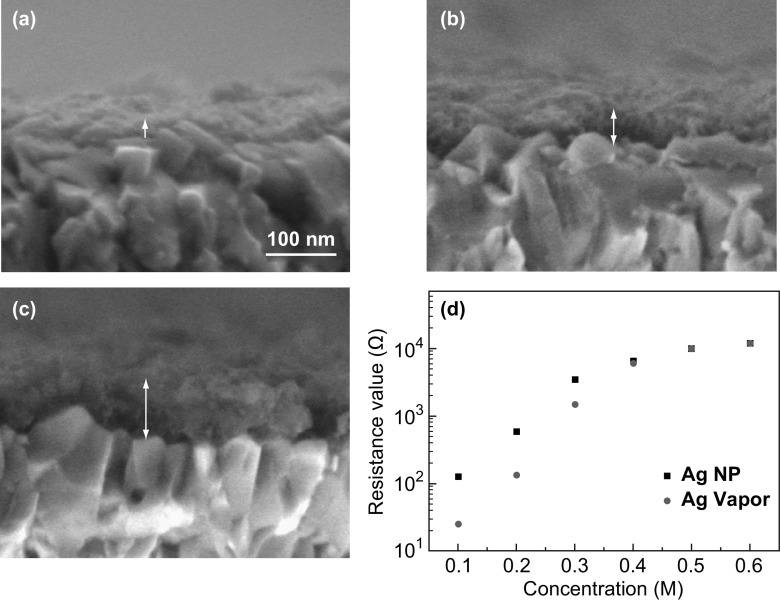
Cross-sectional SEM images of the TiO_2_ films prepared at different concentrations of TiCl_4_ **a** 0.2 M, **b** 0.4 M, **c** 0.6 M. Panels **a**–**c** have the same scale-bar of 100 nm. **d** Average resistance between Ag spots on TiO_2_ layers prepared using different concentrations of TiCl_4_

In general, the thicknesses of the resulting films are affected by the concentration of the precursor solution [31]. Figure 3 shows the cross-sectional SEM images of the TiO_2_ layers formed by SAH using different concentrations of a TiCl_4_ aqueous solution (0.2–0.6 M). The TiO_2_ nanoneedles were well developed on the FTO substrate, forming thin films with thicknesses of 20, 45, and 80 nm for TiCl_4_ aqueous solution concentrations of 0.2, 0.4, and 0.6 M, respectively. The changes in the corresponding surface morphology are shown in Fig. S5. It is well known that the TiO_2_ layer plays an important role in the charge collection within PSCs, where a pinhole-free layer of appropriate thickness is desirable for an effective charge extraction. A simple method to evaluate the density of pinholes within the SAH–TiO_2_ layers is shown in Fig. S6 [32]. The resistance value gradually increased with the TiCl_4_ precursor solution concentration owing to the increased thickness of the TiO_2_ layer. Moreover, the difference between the resistance values for the Ag paste and Ag vapor treatments of the TiO_2_ layers were significantly larger when the TiCl_4_ precursor solution concentration was lower than 0.4 M. This indicated the existence of numerous pinholes. However, when the concentration of the TiCl_4_ precursor solution was 0.4 M or larger, the difference between the resistance values became smaller, implying that an extremely dense TiO_2_ layer was formed.

**Fig. 4:**
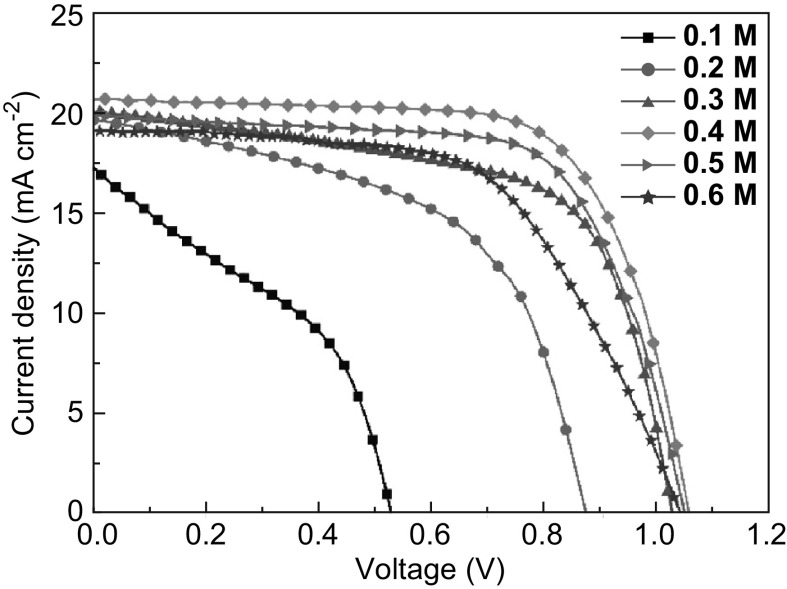
*J*–*V* characteristics of PSCs using different concentrations of TiCl_4_ to prepare the electron transport layer

The current–voltage (*J*–*V*) characteristics of the perovskite cells with different SAH–TiO_2_ film thicknesses obtained by varying the TiCl_4_ precursor solution concentration were obtained to demonstrate the effect of the SAH–TiO_2_ electron transport layers on the efficiency of the perovskite cells; the results are shown in Fig. 4. The detailed parameters are summarized in Table 1. The PCE initially increased and then decreased with the increase of the SAH–TiO_2_ layer thickness. With the 0.1-M TiCl_4_ precursor solution, the coverage of TiO_2_ on the FTO substrate was unsatisfactory (Fig. S5a), which increased the risk of contact between the FTO substrate and perovskite, leading to a low photovoltaic performance. With the increase of the concentration of the TiCl_4_ precursor solution to 0.4 M, the coverage on the FTO substrate improved and pinholes gradually disappeared (Figs. S5b–d and 3d). Therefore, all three photovoltaic device parameters, short-circuit current density (*J*_sc_), open-circuit potential (*V*_oc_), and fill factor (FF), increased during this evolution process, suggesting an efficient suppression of the charge recombination on the FTO surface. The SAH–TiO_2_ films (~ 45 nm) prepared by treating the FTO substrate with 0.4 M TiCl_4_ exhibited the highest PCE of 17.09% with a *J*_sc_ of 21.96 mA cm^−2^, *V*_oc_ of 1.069 V, and FF of 0.728. Furthermore, the integrated photocurrent from the incident photon-to-current conversion efficiency was 21.91 mA cm^−2^, which is close to that from the *J*–*V* measurement (Fig. S8). However, a further increase in the concentration of TiCl_4_ led to a deterioration of the device performance owing to the lower electron transport in the thicker TiO_2_ layers and prolonged transport path, increasing the series resistance [33]. This could be confirmed by the apparent decrease in the *J*_sc_ and FF values.

**Table 1:** Photovoltaic parameters of SAH–TiO_2_-based PSCs fabricated using the SAH method with different concentrations of TiCl_4_

TiCl_4_ concentration (M)	*J*_sc_ (mA cm^−2^)	*V*_oc_ (V)	FF	PCE (%)
0.1	18.37	0.686	0.435	5.48
0.2	20.61	0.985	0.614	12.46
0.3	21.13	1.032	0.678	14.78
0.4	21.96	1.069	0.728	17.09
0.5	20.84	1.057	0.703	15.48
0.6	19.91	1.043	0.667	13.87

**Fig. 5:**
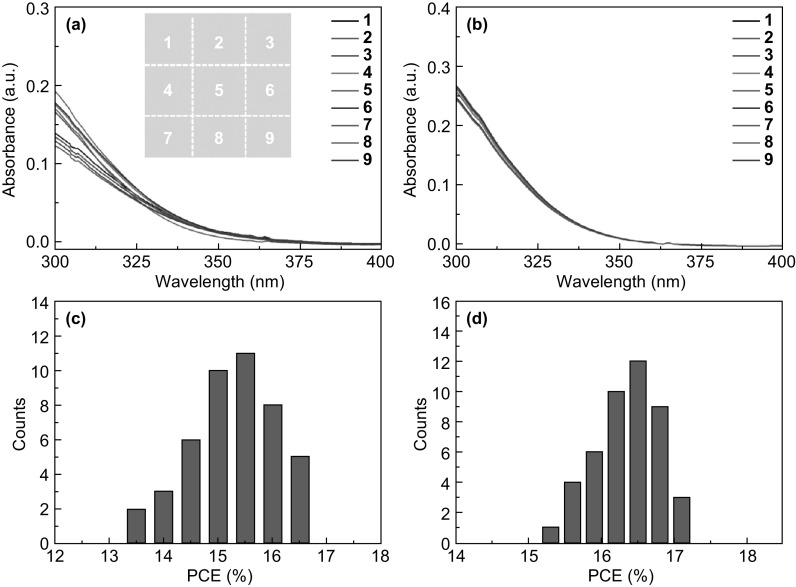
UV–Vis light absorption spectra of 9 pieces of 2 × 2 cm^2^ TiO_2_ films obtained by **a** SC, **b** SAH. PCE distribution histogram of 45 **c** SC–TiO_2_-based PSCs, **d** SAH–TiO_2_-based PSCs. The films and PSCs were fabricated on the 9 sub-areas divided along the white dashed lines on the SC and SAH TiO_2_ films (6 × 6 cm^2^)

It is well known that SC is only suitable for a small-area deposition simply, as the spinning rates at the core and edges significantly differ, leading to a poor uniformity of the layers in a large-area deposition. To verify this, we attempted to deposit a TiO_2_ layer on a large-area substrate (approximately 6 × 6 cm^2^) by SC; a reference sample prepared by SAH was used for comparison. The large coated substrates were divided into 9 small pieces (2 × 2 cm^2^) along the white dashed lines shown in the inset of Fig. 5a. The UV–Vis light absorption spectra of these TiO_2_ films deposited by SC and SAH are shown in Fig. 5a, b, respectively. The light absorption at 320 nm was chosen as the index to determine the film uniformity as the absorption was proportional to the TiO_2_ film thickness. The variation in the absorption of the SAH–TiO_2_ film at 320 nm was only 6%, whereas that of the SC–TiO_2_ film was 17%, which suggested that SAH was a more efficient deposition system than SC to fabricate uniform TiO_2_ films. Undoubtedly, the uniformity of the TiO_2_ layer influences the device performance. To illustrate this point, perovskite devices were fabricated with small TiO_2_ pieces obtained, as mentioned above. The statistical PCE distributions of 45 samples in 5 batches of PSCs based on SC–TiO_2_ and SAH–TiO_2_ films are shown in Fig. 5c, d, respectively. The PCE distribution of the SC–TiO_2_ PSCs was broader, varying from 13.56 to 16.63%, with an average of 15.27%. In contrast, the PCE distribution of the SAH–TiO_2_ PSCs was narrower (15.31–17.12%), with an average efficiency of 16.34%. Detailed information is shown in Fig. S9 and Table S1. The fluctuations in PCE for the SAH–TiO_2_-based PSCs in different sub-areas were very small, indicating a higher reproducibility.

**Fig. 6:**
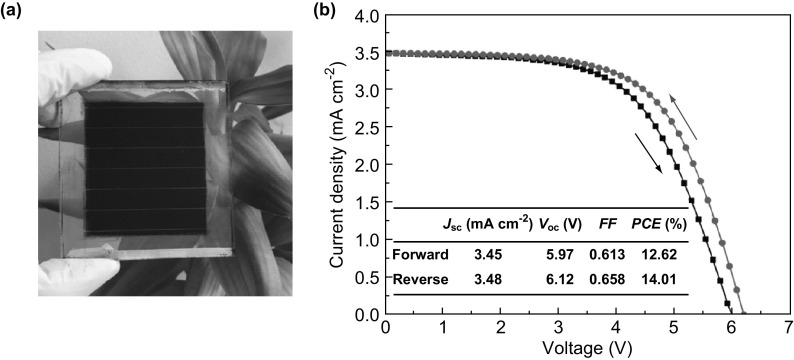
**a** Photograph of the backside of the perovskite module consisting of 6 sub-cells with a total aperture area of 16 cm^2^. **b** *J*–*V* curves of the solar module measured in forward and reverse modes under a simulated solar light of AM 1.5 G and 100 mW cm^−2^

The proposed SAH method for TiO_2_ film growth exhibited a remarkable uniformity over large surface areas. A 4 × 4 cm^2^ sub-module comprising 6 cells connected in series was considered to demonstrate the consistency of the SAH method. A photograph of the module and its *J*–*V* characteristics are shown in Fig. 6. The module exhibited *V*_oc_ of 6.12 V, *J*_sc_ of 3.48 mA cm^−2^, remarkably high FF of 0.658, and overall conversion efficiency of 14.01% with hysteresis. The obtained results of the module using SAH–TiO_2_ as the electron transport layer are encouraging for the development of perovskite photovoltaic technologies at low temperatures.

## Conclusion

A simple SAH method was introduced for large-scale deposition of TiO_2_ films at low temperatures. Using this method, we achieved compact homogeneous TiO_2_ films with a needle-like morphology. A solar module fabricated using SAH–TiO_2_ films exhibited a PCE of 14.01% with hysteresis. The results indicated that SAH is a convenient and versatile novel approach for the deposition of large-area TiO_2_ films, demonstrating a large potential for practical applications in the future.

## Electronic supplementary material

Below is the link to the electronic supplementary material.

Supplementary material 1 (PDF 652 kb)
