# Supplementary material for: Low-Temperature Soft-Cover-Assisted Hydrolysis Deposition of Large-Scale TiO2 Layer for Efficient Perovskite Solar Modules
Source: Nanomicro Lett. 2018 Apr 30;10(3):49. doi: 10.1007/s40820-018-0203-7 (PMC6199095; doi:10.1007/s40820-018-0203-7)
Supplement: Supplementary file 1 — Supplementary material 1 (PDF 652 kb) [file 40820_2018_203_MOESM1_ESM.pdf]

Supporting Information for

Low-Temperature Soft-Cover-Assisted Hydrolysis Deposition of Large-Scale TiO_2 Layer for Efficient Perovskite Solar Modules

Jinjin He[1], Enbing Bi[1], Wentao Tang[1], Yanbo Wang[1], Xudong Yang[1, 2], Han Chen[1, 2,]*, Liyuan Han[1,]*

[1]State Key Laboratory of Metal Matrix Composites, School of Material Science and Engineering, Shanghai Jiao Tong University, 800 Dong Chuan Road, Shanghai 200240, People's Republic of China

[2]Materials Genome Initiative Center, Shanghai Jiao Tong University, 800 Dong Chuan Road, Shanghai 200240, People's Republic of China

*Corresponding authors. E-mail: chen.han@sjtu.edu.cn (Han Chen); han.liyuan@sjtu.edu.cn (Liyuan Han)

Supplementary Figures and Table

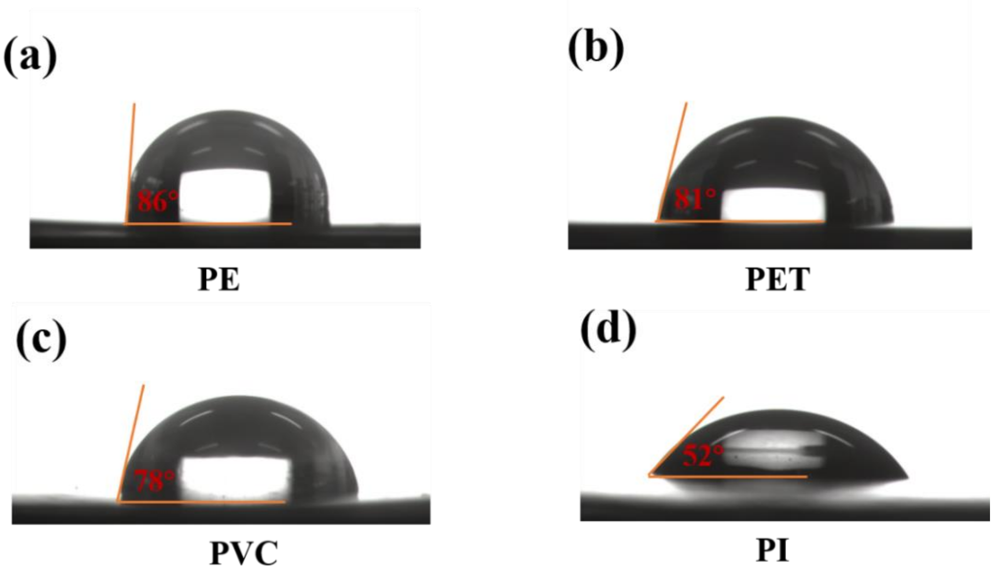

Fig. S1 Contact angles of water on different soft films

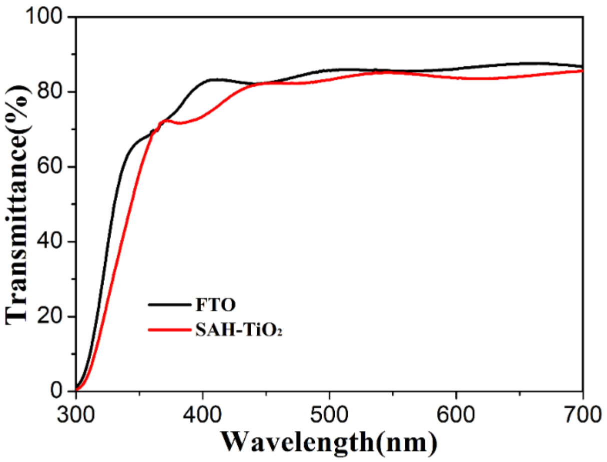

Fig. S2 Transmittance spectra of FTO substrate and SAH-TiO_2 films deposited on FTO substrate

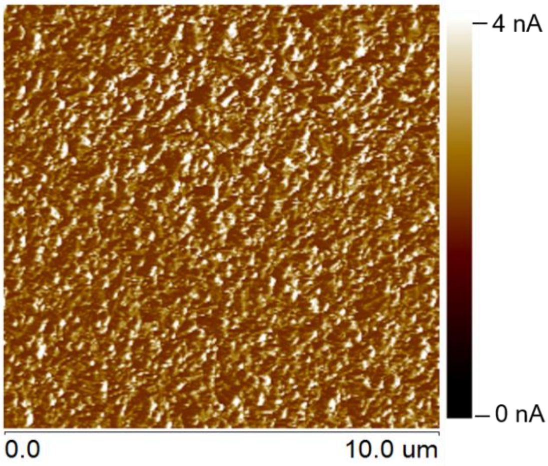

Fig. S3 The conductivity mapping result for TiO_2 films

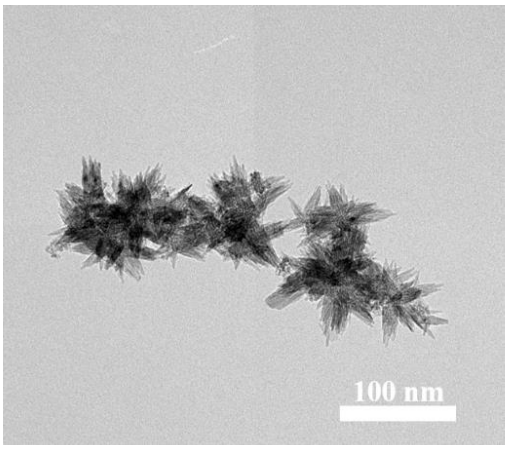

Fig. S4 TEM image of the TiO_2 material scratched from the TiO_2 film

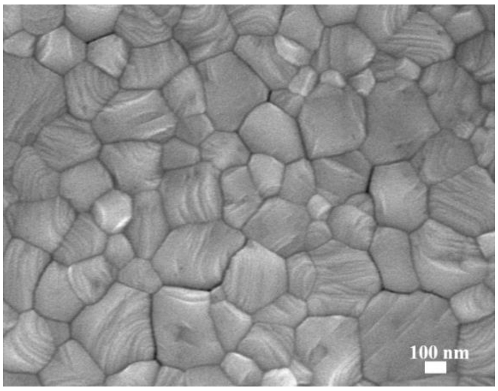

Fig. S5 SEM images of perovskite film based on SAH-TiO_2 layer

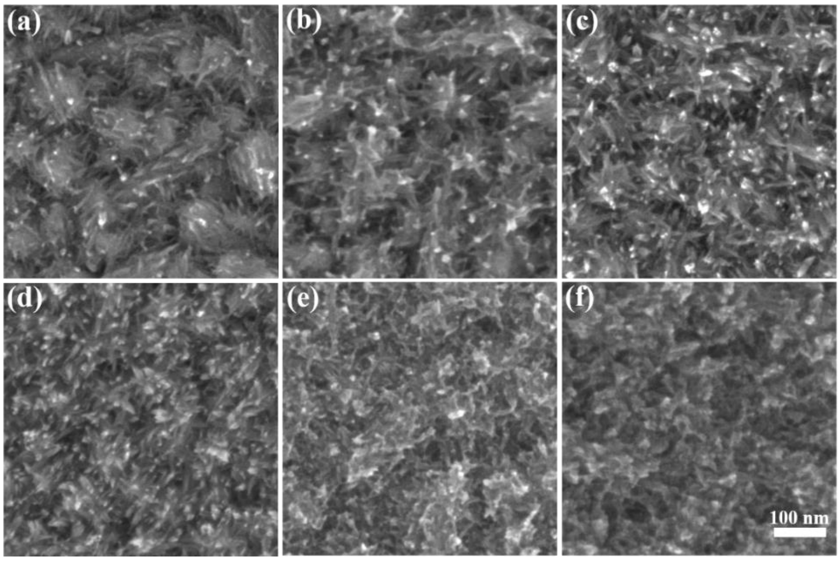

Fig. S6 SEM images of the TiO_2 films prepared using different concentrations of $TiCl_4$ precursor solution at **a** 0.1 M, **b** 0.2 M, **c** 0.3 M, **d** 0.4 M, **e** 0.5 M, and **f** 0.6 M. Panels a−f have the same scale bar of 100 nm

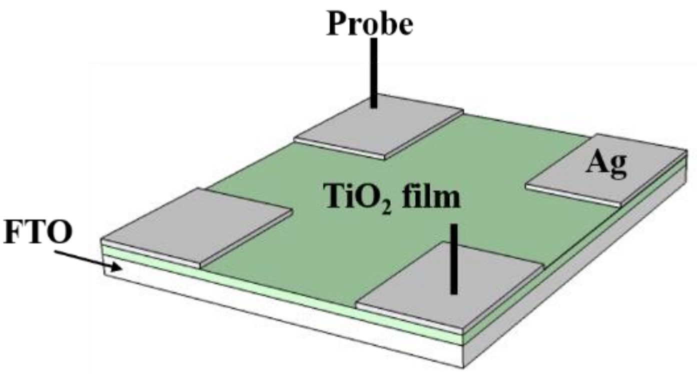

Fig. S7 Schematic of the resistance measurements in TiO_2 layers after coating with Ag paste and vacuum-evaporated Ag

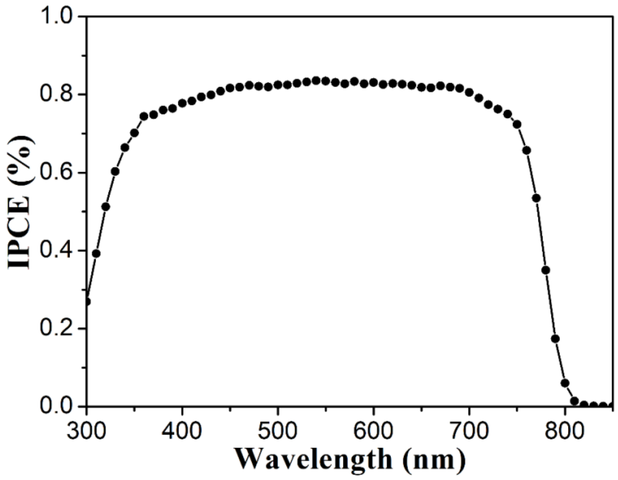

Fig. S8 External quantum efficiency spectra for the devices with SAH-TiO_2 films prepared by treating the FTO substrate with 0.4 M $TiCl_4$

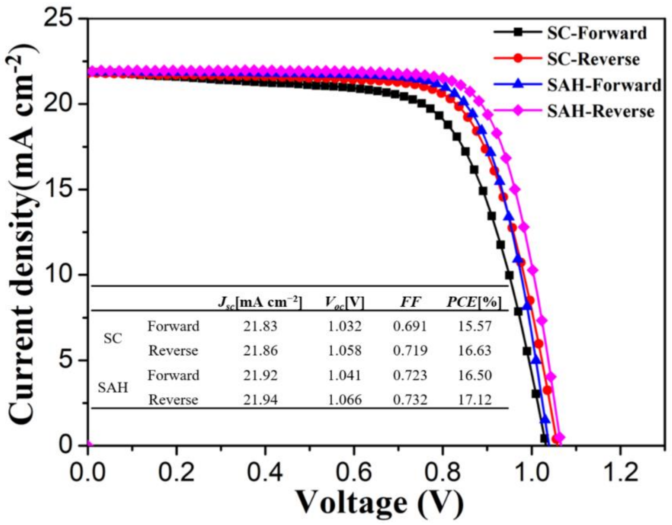

Fig. S9 $J-V$ curves of best PSCs based on SC-TiO_2 and SAH-TiO_2 films in forward and reverse scans

Table S1 Photovoltaic properties of PSCs based on SC-TiO_2 and SAH-TiO_2 films[a]

	J_{sc} (mA cm^{-2})	V_{oc} (V)	FF	PCE (%)
SC	21.15±0.8	1.042±0.02	0.693±0.03	15.27±0.36
SAH	21.43±0.6	1.053±0.02	0.724±0.01	16.34±0.8

[a]Photovoltaic data is average values of 45 cells. Cell area=1.02 cm^2
